# Controlling Microdroplet Inner Rotation by Parallel Carrier Flow of Sesame and Silicone Oils

**DOI:** 10.3390/mi13010009

**Published:** 2021-12-22

**Authors:** Hibiki Yoshimura, Daiki Tanaka, Masahiro Furuya, Tetsushi Sekiguchi, Shuichi Shoji

**Affiliations:** 1 Major in Nanoscience and Nanoengineering, Waseda University, 3-4-1 Okubo, Shinjuku, Tokyo 169-8555, Japan; shojis@waseda.jp; 2 Research Organization for Nano & Life Innovation, Waseda University, 513 Tsurumakicho, Shinjuku, Tokyo 162-0041, Japan; d.tanaka@ruri.waseda.jp (D.T.); t-sekiguchi@waseda.jp (T.S.); 3 Cooperative Major in Nuclear Energy, Graduate School of Advanced Science and Engineering, Waseda University, 3-4-1 Okubo, Shinjuku, Tokyo 169-8555, Japan; furuya@aoni.waseda.jp

**Keywords:** microdroplets, passive, rotation, parallel flow

## Abstract

We developed a method for passively controlling microdroplet rotation, including interior rotation, using a parallel flow comprising silicone and sesame oils. This device has a simple 2D structure with a straight channel and T-junctions fabricated from polydimethylsiloxane. A microdroplet that forms upstream moves into the sesame oil. Then, the largest flow velocity at the interface of the two oil layers applies a rotational force to the microdroplet. A microdroplet in the lower oil rotates clockwise while that in the upper oil rotates anti-clockwise. The rotational direction was controlled by a simple combination of sesame and silicone oils. Droplet interior flow was visualized by tracking microbeads inside the microdroplets. This study will contribute to the efficient creation of chiral molecules for pharmaceutical and materials development by controlling rotational direction and speed.

## 1. Introduction

Microdroplets have been used as efficient platforms for chemical and biological experiments. Many research groups have reported that samples can be individualized and controlled in small volumes within microdroplets [1,2,3,4,5]. In recent years, the rotation of microdroplets has been attracting a lot of attention because the rotation of samples is an important factor in the efficient mixing of chemicals, enhancing chemical synthesis, and detecting biochemicals [6,7]. For various applications employing both microdroplets and rotation characteristics, a method of rotating microdroplets is required.

Several methods of rotating droplets have been reported, which can roughly be classified into active and passive types, according to the rotation technique. In active methods, extra energy fields are introduced to the experimental platform for controlling microdroplets within a flow. Most methods reported to date have been active [8,9]. For example, Gires et al. [10] reported synchronizing the internal solution with an external magnetic field, while Chabreyrie et al. [11] used resonances between the natural frequencies of the microdroplets and those of the external force. These methods have been successful in controlling the inner flow accurately. However, active methods using an external physical field are not applicable for the static chemical reactions needed for most common syntheses. This is because excessive energy from extra energy fields induces damage in chemical samples. Moreover, active methods are necessary for complex experimental systems, and advanced techniques are needed for handling such systems.

In contrast, passive methods are achieved by only microdroplet movement. The microdroplet inner flow is controlled by the device structure or the flow conditions of carrier liquids [12]. Some analyses of microdroplet inner flow have been reported [13,14,15]. Wang et al. [16] reported droplet inner convection in straight and winding channels. Song et al. [17] showed an experimental test of a simple argument that predicts droplet inner flow. These studies have contributed to enhanced mixing. Moreover, passive droplet rotational methods are rarely reported. To enhance mixing or obtain a specific inner flow, more complex channel structures may be required. Such channel structures are difficult to apply for chemicals and biochemicals because advanced flow-control techniques and complex fabrication processes are needed. However, an advantage of a passive method is that various samples can be used without extra energy field effects. Therefore, we developed a new passive method for microdroplet inner rotation in a simple device that uses two parallel carrier flows. In addition, we demonstrated a simple technique for controlling the rotational direction.

## 2. Principle

**Figure 1:**
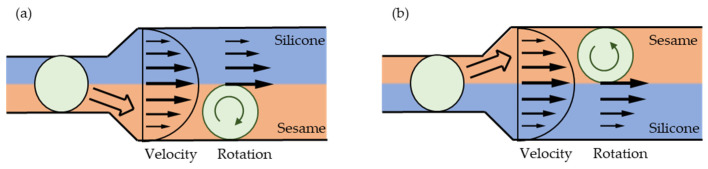
Schematic view of microdroplet rotation inside oil flow in a straight channel. (**a**) Clockwise rotation; (**b**) anti-clockwise rotation.

Figure 1 shows a schematic view of a microdroplet inside a straight channel with flow. The flow can be provided by a single carrier oil, or two different carrier oils can be used to create parallel flow. In carrier flow, the velocity at the center of channel is larger, while the velocity near the channel wall is smaller.

If the water droplet moves into the lower layer in a parallel carrier flow, a clockwise rotational force is generated (Figure 1a) because the velocity at the upper interface of the microdroplet is larger than that at the lower interface. If the water droplet moves into the upper layer, an anti-clockwise rotational force is generated (Figure 1b) because the velocity at the lower interface of the microdroplet is larger than that at the upper interface.

The water repellency of each oil determines whether a water droplet moves into the upper or lower oil in a parallel flow. If the water droplet moves to the boundary (center) between two parallel flows, the water droplet is forced away from the oil with a greater water repellency. The water repellency of silicone oil is larger than that of sesame oil. Therefore, the water droplet is pushed out of the silicone oil and moves into the sesame oil. Thus, in the parallel flow system, the rotational direction is controlled by selecting which oils form the upper and lower layers. We developed a new simple device for implementing this system.

## 3. Device Design and Fabrication

**Figure 2:**
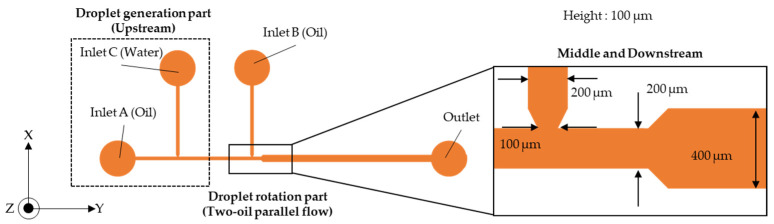
Schematic view and size details of the microdroplet rotation device.

A schematic view of the size and configuration of the microdroplet rotation device is shown in Figure 2. Two T-junctions are connected in series to a straight channel that widens. This device allows for two types of flow, namely, clockwise rotation (Figure 1a) and anti-clockwise rotation (Figure 1b). In clockwise rotation, water droplets are introduced from the upstream T-junction (Inlet C), and sesame oil is introduced in the X-direction from the far-left inlet (Inlet A). To generate two parallel flows, silicone oil is introduced from the middle T-junction (Inlet B). In the downstream straight channel, sesame and silicone oil form a two-oil parallel carrier flow. This flow induces water droplet rotation in the upper or lower layer of this channel. The rotational direction is controlled by the simple combination of sesame and silicone oil. When sesame oil is introduced from the inlet A, sesame oil is the lower layer. In anti-clockwise rotation, sesame oil is introduced from inlet B, and sesame oil is the upper layer.

The width of the upstream and middle T-junctions is 200 µm. The width of the downstream straight channel is 400 µm. To control a droplet in the upper or lower carrier flow, the width of the downstream channel widens to twice the width of the middle channel. The height of the structure is about 100 µm.

This device was fabricated by soft lithography. The channel mold was formed with SU-8 photoresist. Polydimethylsiloxane (PDMS) was poured into the mold, thermally cured, and peeled from the mold to form the PDMS device structure. The structure was bonded to a PDMS-coated glass substrate by plasma treatment (Aiplasma, Matsushita Electric Works).

## 4. Results and Discussion

### 4.1. Experimental Setup and Materials

All liquids were introduced by syringes (1750CX, Hamilton, Reno, NV, USA) and syringe pumps (Legato 101, KD Scientific, Holliston, MA, USA). The experimental result was observed by a microscope (IX71, Olympus, Tokyo, Japan) and high-speed camera (FASTCAM Mini Ax, Photoron, Tokyo, Japan). Silicone oil (KF-96-200cs, Shin-Etsu Chemical, Tokyo, Japan) and sesame oil (Zyunsei sesame oil, Kadoya, Tokyo, Japan) were used for the carrier oils. Water was used for the microdroplets. To visualize the inner flow, water droplets were infused with microbeads (Cat. No. 15715-5, Polysciences, Warrington, PA, USA), 6 µm in diameter.

### 4.2. Rotational Direction

This section explains the rotational directions of microdroplets. Flow rates of sesame or silicone oil were fixed at 2.0 µL/min. The water flow rate was 1.2 µL/min.

**Figure 3:**
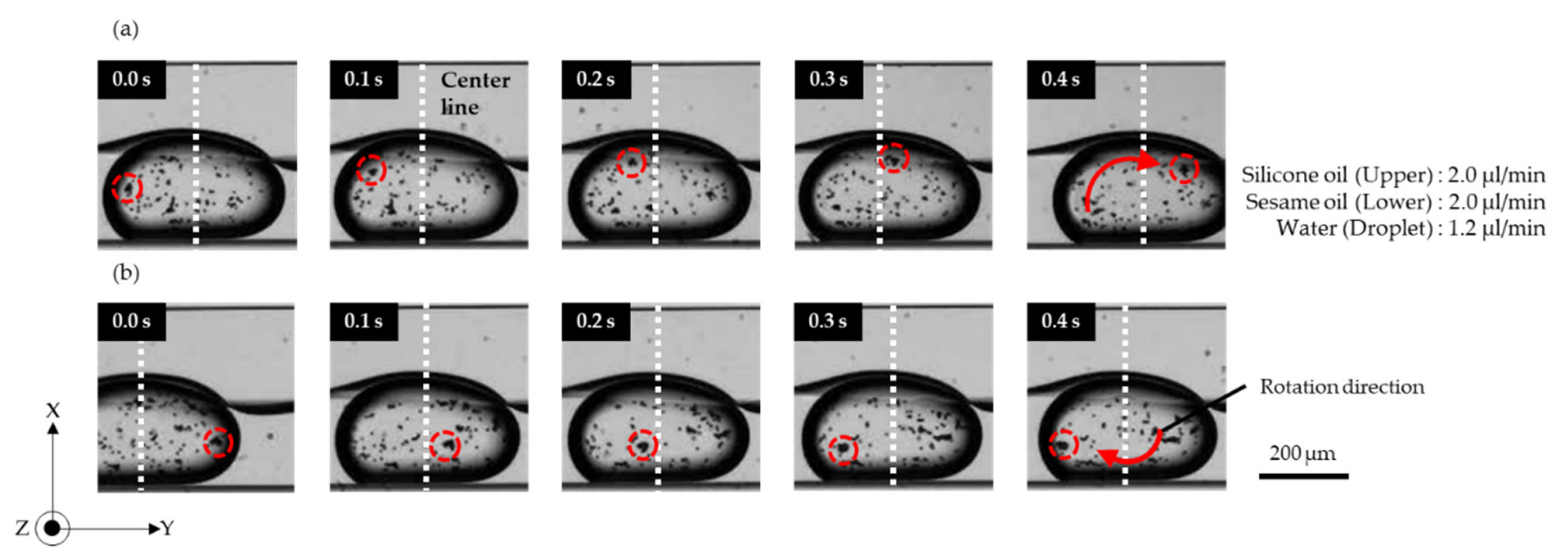
Images of clockwise rotation of a microdroplet. (**a**) Movement of upper beads in the droplet; (**b**) movement of lower beads in the droplet.

Figure 3 (Supplementary Material, Video S1) shows the clockwise rotation of a microdroplet. Sesame oil was introduced from inlet A. Water droplets were introduced from inlet C into the sesame oil. The two-oil parallel flow was formed by injecting silicone oil from inlet B. The beads, located close to the upper interface, traveled in the flow field of the lower layer (Figure 3a). In contrast, beads that reached the lower interface traveled in the direction opposite to the outer flow field (Figure 3b).

**Figure 4:**
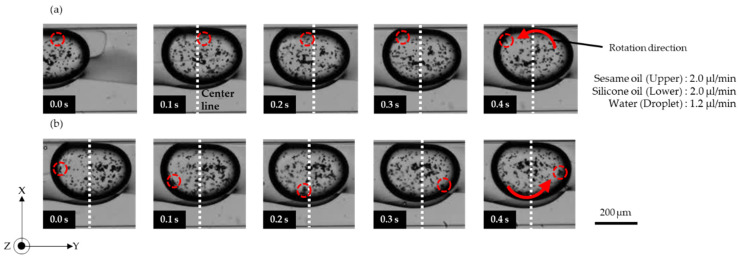
Images of anti-clockwise rotation of a microdroplet. (**a**) Upper beads in the droplet; (**b**) lower beads in the droplet.

Figure 4 (Supplementary Material, Video S2) shows that the microdroplet could also be rotated anti-clockwise by simply reversing the injection locations for silicone oil and sesame oil; that is, silicone oil was introduced from inlet A, and sesame oil was introduced from inlet B. Water droplets were generated from inlet C into the silicone oil layer. The microdroplets moved to the sesame oil layer at inlet B owing to the differential water repellency of silicone and sesame oils. The beads close to the upper interface traveled in the direction opposite to the outer flow field (Figure 4a). In contrast, beads that reached the lower interface traveled in the same flow field as the upper layer (Figure 4b).

In both clockwise (Supplementary Material, Video S1) and anti-clockwise (Supplementary Material, Video S2) rotations, it was also observed that the beads between the upper and lower interfaces of the droplet traveled in the direction opposite to the outer flow field. These inner flows were considered to be an effect in the Z-direction. The velocity near the channel wall is small in the Z-direction, as is the channel wall in the Y-direction. If the droplet diameter is large, the droplet is considered to be touching the Z-direction channel walls. Therefore, the beads close to the Y- and Z-direction channel walls traveled in the opposite direction to the outer flow field. In this paper, we focused on the droplets inner rotational flow near the middle of the Z-direction, where the effects of the Z-direction channel walls were very small.

Consequently, the rotation was achieved by the parallel flow of two different oils. Moreover, we succeeded in controlling the microdroplet rotational direction with a simple combination of silicone oil and sesame oil.

### 4.3. Plot of Microdroplet Inner Flow and Rotational Speed

This section discusses the trajectory of the droplet inner rotational flow and rotational speed. The flow rate of water was fixed at 1.2 µL/min.

**Figure 5:**
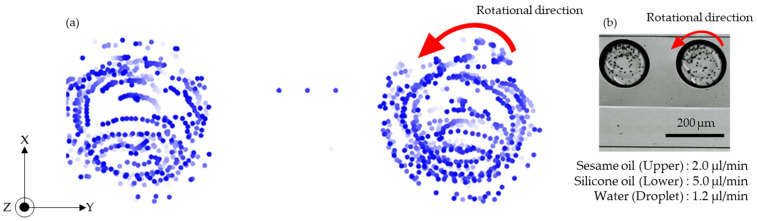
Trajectory of anti-clockwise rotational flow in microdroplets. (**a**) Beads plotted against time; (**b**) image of the droplet rotating anti-clockwise.

Figure 5a shows the trajectory of the inner droplet flow. The upper layer was sesame oil (2.0 µL/min), and the lower layer was silicone oil (5.0 µL/min). Figure 5b (Supplementary Material, Video S3) is an image of the anti-clockwise droplet rotation and was created through image analysis. The MATLAB function ‘regionprops’ detects the center location of each bead in an acquired image. The plots are rendered with various shades of blue according to the observation time. Beads in the early stage of observation are plotted in light blue, and beads in the end stage of observation are plotted in dark blue. The carrier oil moved the droplet and beads from left to right. In this analysis, the beads are plotted with compensation for the component of movement in the X-direction caused by the carrier oil flow. The beads close to the droplet–oil interface moved along the interface in an anti-clockwise direction. Moreover, the beads close to the center of the droplet also moved in a smaller circle in the anti-clockwise direction. This is shown by the color of plots shifting from light blue to dark blue in the anti-clockwise direction.

The anti-clockwise rotational droplets were introduced into the upstream silicone oil. When the silicone oil flow rate increased, the diameter of droplets decreased to about 180 µm. The shape of microdroplets was suggested to be close to a sphere because the droplet shape in the 2D images was a circle, and the diameter of the inlet channel was 100 µm. This was thought to reduce the effect on the microdroplets from the channel wall in the Z-direction. The microdroplets were affected by the channel wall in the Y-direction and by the carrier flow in the X-direction. Then, the microdroplet inner flow approached rotation in the X- and Y-directions.

**Figure 6:**
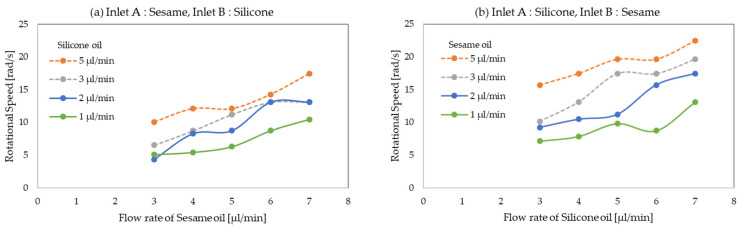
Rotational speed of droplet versus carrier oil flow rate, when the droplet. diameter was varied accordingly. (**a**) Clockwise rotation; (**b**) anti-clockwise rotation.

**Figure 7:**
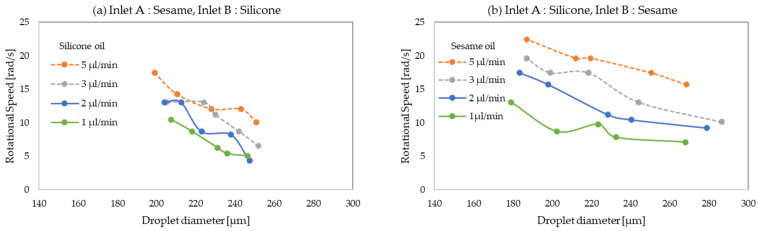
Rotational speed of droplet versus droplet diameter. (**a**) Clockwise rotation; (**b**) anti-clockwise rotation.

The droplet rotational speed was calculated from the distance traveled by the plots. Figure 6 shows the change in rotational speed against the flow rate of the carrier oils. In this experiment, the flow rate of water was fixed (1.2 µL/min) and the flow rate of carrier oils was changed. The droplet diameter was varied accordingly. The relationship between the rotation speed and droplet diameter is shown in Figure 7.

In Figure 6a, the clockwise rotational speed increased with the increase in the sesame oil flow rate when the silicone oil flow rate was fixed. Furthermore, the rotational speed also tended to increase when the silicone oil flow rate was increased. The rotational force was generated by the large flow at the interface of the silicone and sesame oils. The large flow was caused by both oils, and the rotational speed was considered to be controlled by both carrier oil flow rates. The maximum clockwise rotational speed (sesame oil = 7 µL/min; silicone oil = 5 µL/min) was about 4 times the minimum (sesame oil = 3 µL/min; silicone oil = 2 µL/min).

As shown in Figure 6b, the anti-clockwise rotational speed also increased with increasing silicone and sesame oil flow rates. The maximum anti-clockwise rotational speed (silicone oil = 7 µL/min; sesame oil = 5 µL/min) was about 4.5 times the minimum (silicone oil = 3 µL/min; sesame oil = 1 µL/min). Multiple rotational speeds were achieved with different combinations of carrier oil flow rates.

Consequently, it was suggested that the droplet rotational speed can be controlled by the flow rate of both silicone and sesame oil.

Figure 7 shows the rotational speed against the droplet diameter. The flow rate of the water and carrier oils were the same as in Figure 6. As shown in Figure 7a, the clockwise rotational speed increased with the decrease in droplet diameter. In Figure 7b, the anti-clockwise rotational speed also increased with the decrease in droplet diameter. The droplet was formed in upstream oil (clockwise: sesame oil; anti-clockwise: silicone oil). Therefore, when the flow rate of upstream oil increased, the droplet rotational speed increased, and the droplet diameter decreased. This suggests that the appropriate combination of rotational speed and droplet diameter can be selected by the choice of carrier oils.

## 5. Conclusions

We developed a simple and new method for the passive rotation of hydrous microdroplets in oil. A two-oil parallel carrier flow was used to achieve rotational flow within the microdroplets. Furthermore, we successfully controlled the droplet rotational direction by a simple combination of sesame and silicone oils, which have different hydrophobic properties. The inner rotational flow was visualized by plotting the movement of beads within the droplets. The rotational flow was generated in the entire droplet, and the center of rotation was near the interface of the two oils rather than the droplet center. It was suggested that the droplet rotational speed could be controlled by adjusting the carrier oil flow rate.

In this study, the experimental system consisted of a simple channel structure and three different fluids. It was not necessary to apply an external force for advanced control of droplet rotation. The proposed device structure can be integrated into PDMS systems with other experimental elements. Therefore, we believe that this study will contribute to droplet-based chemical and biochemical applications.

## Supplementary Materials

The following supporting information can be downloaded at: https://www.mdpi.com/article/10.3390/mi13010009/s1, Video S1: clockwise rotation of a microdroplet, Video S2: anti-clockwise rotation of a microdroplet, Video S3: original movie of anti-clockwise rotational droplet trajectory.
